# Chimerism-Based Tolerance to Kidney Allografts in Humans: Novel Insights and Future Perspectives

**DOI:** 10.3389/fimmu.2021.791725

**Published:** 2022-01-05

**Authors:** Manuel Alfredo Podestà, Megan Sykes

**Affiliations:** ^1^ Renal Division, ASST Santi Paolo e Carlo, Department of Health Sciences, University of Milan, Milano, Italy; ^2^ Columbia Center for Translational Immunology, Department of Medicine, Department of Surgery, Department of Microbiology and Immunology, Columbia University, New York, NY, United States

**Keywords:** chimerism and tolerance, kidney, transplantation, mixed chimerism, clinical protocol

## Abstract

Chronic rejection and immunosuppression-related toxicity severely affect long-term outcomes of kidney transplantation. The induction of transplantation tolerance – the lack of destructive immune responses to a transplanted organ in the absence of immunosuppression – could potentially overcome these limitations. Immune tolerance to kidney allografts from living donors has been successfully achieved in humans through clinical protocols based on chimerism induction with hematopoietic cell transplantation after non-myeloablative conditioning. Notably, two of these protocols have led to immune tolerance in a significant fraction of HLA-mismatched donor-recipient combinations, which represent the large majority of cases in clinical practice. Studies in mice and large animals have been critical in dissecting tolerance mechanisms and in selecting the most promising approaches for human translation. However, there are several key differences in tolerance induction between these models and humans, including the rate of success and stability of donor chimerism, as well as the relative contribution of different mechanisms in inducing donor-specific unresponsiveness. Kidney allograft tolerance achieved through durable full-donor chimerism may be due to central deletion of graft-reactive donor T cells, even though mechanistic data from patient series are lacking. On the other hand, immune tolerance attained with transient mixed chimerism-based protocols initially relies on Treg-mediated suppression, followed by peripheral deletion of donor-reactive recipient T-cell clones under antigenic pressure from the graft. These conclusions were supported by data deriving from novel high-throughput T-cell receptor sequencing approaches that allowed tracking of alloreactive repertoires over time. In this review, we summarize the most important mechanistic studies on tolerance induction with combined kidney-bone marrow transplantation in humans, discussing open issues that still need to be addressed and focusing on techniques developed in recent years to efficiently monitor the alloresponse in tolerance trials. These cutting-edge methods will be instrumental for the development of immune tolerance protocols with improved efficacy and to identify patients amenable to safe immunosuppression withdrawal.

## Introduction

Renal transplantation is the established treatment of choice for kidney failure, as it confers both the highest survival and the best quality of life compared to other renal replacement therapies (1). Despite continuous advances in the field of solid organ transplantation, long-term outcomes of kidney allografts have only modestly improved in the last decades. Immunosuppressive therapies consistently control acute rejection, but have little effect on chronic rejection, which leads to graft loss in 50% of cases at 10 years (2). In addition, approximately half of the kidney transplants lost are due to death with a functioning graft: the impact of chronic immunosuppression has potentially devastating consequences in terms of cardiovascular disease, infection and malignancy (3–5), and may severely impair recipients’ quality of life.

The induction of tolerance, i.e. the lack of destructive immune responses to a transplanted organ in the absence of immuno-suppression, could potentially overcome both of these limitations. Tolerance in kidney transplantation can be functionally defined by stable renal function and absence of histologic, immune and molecular signs of rejection on a kidney biopsy obtained after complete withdrawal of immunosuppression for at least one year. Spontaneous tolerance is unfortunately a rare and unpredictable event that has been described in a small minority among the patients who choose to discontinue their immunosuppression, who retained graft function despite complete withdrawal of immunosuppression (6).

Among the different methods used to induce tolerance in animal models of kidney transplantation, few have been successfully translated to clinical application. Those protocols that have succeeded in patients entail combined kidney and bone marrow transplantation (CKBMT) as a strategy to induce chimerism, a state wherein donor hematopoietic cells engraft into the recipient bone marrow at a level sufficient to be detected by conventional (as opposed to sensitive PCR-based) methods.

Three centers have developed clinical CKBMT protocols, one of which has so far succeeded in achieving tolerance only in the HLA-identical transplant setting (7). Investigators from Stanford University used total lymphoid irradiation combined with anti-thymocyte globulin to facilitate the engraftment of donor hematopoietic stem cells (HSC), which were infused along with a fixed number of donor T cells after kidney transplantation. Mixed chimerism persisting for at least 6 months was achieved in 83% of the 29 HLA-matched patients treated with this protocol. Mixed chimerism was consistently associated with a tolerant state that allowed safe withdrawal of immunosuppression. Unfortunately, when a similar protocol was applied to haplotype-matched donor-recipient pairs, immunosuppressive drug weaning below therapeutic levels led to loss of chimerism and rejection episodes (8, 9).

Only two strategies have succeeded in effectively inducing operational tolerance across HLA barriers so far. As HLA mismatches are commonly present in solid organ transplantation, in this review we will discuss the features of these regimens and the novel mechanistic insights offered by recent studies in the field.

## Chimerism-Based Protocols for Tolerance Induction Across MHC Barriers

### Full Donor Chimerism

**Figure 1:**
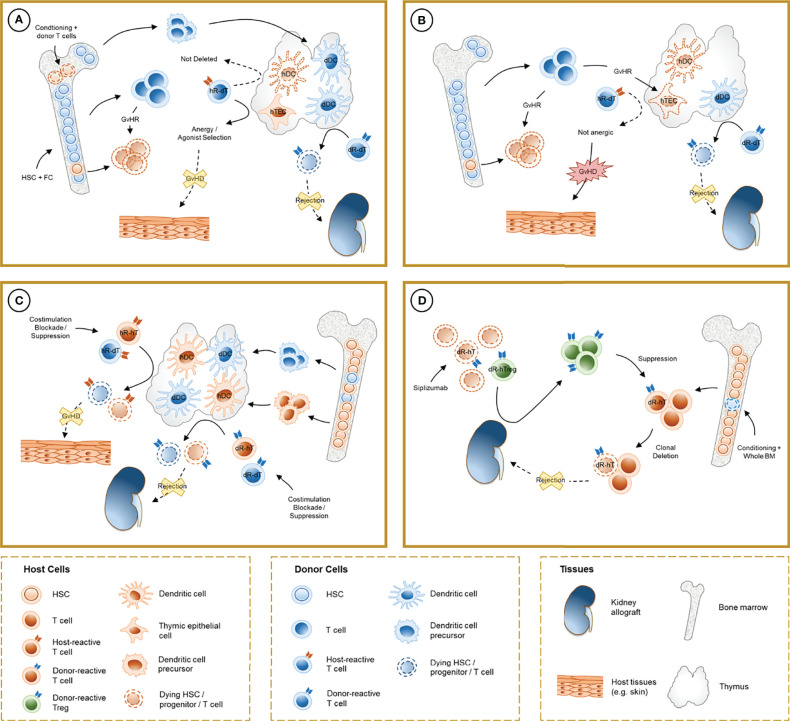
Schematic representation of the mechanisms involved in chimerism-based tolerance to kidney allografts. **(A)** The induction of full-donor chimerism through hematopoietic stem cell (HSC) infusion along with facilitating cells (FC) after non-myeloablative conditioning results in destruction of host HSC, presumably by graft-versus-host reaction (GvHR) from infused donor T cells, and durable engraftment of donor hematopoietic precursors. After thymic repopulation by donor-derived dendritic cells (dDC), donor-reactive T cells from the donor (dR-dT) undergo clonal deletion in the thymus (central tolerance). Host-reactive donor T cells (hR-dT) are incompletely deleted, reflecting the absence of a self-renewing source of recipient APCs, but functional tolerance to the recipient may be achieved by a combination of mechanisms (anergy and selection of host-specific Tregs) that involve recipient thymic epithelial cells (hTEC). **(B)** Destruction of hTEC and thymic structure by GvHR may cause failure of negative selection and precipitate graft-versus-host disease (GVHD). **(C)** In durable mixed chimerism, donor-derived precursors populate the host thymus and differentiate into DC (dDC) without depletion of their host-derived counterparts (hDC). Donor- and host-reactive T cells from both the donor and the host undergo negative selection, allowing allograft tolerance without GVHD. Treg-mediated suppression may also play a role in experimental regimens where clonal deletion is incomplete. **(D)** In CKBMT patients receiving a siplizumab-based conditioning regimen and unprocessed bone marrow, transient mixed chimerism promotes peripheral tolerance. Host Tregs are relatively spared from global T cell depletion, and donor-reactive host Tregs (dR-hTreg) are expanded by antigenic pressure from the graft. Emerging donor-reactive T cells, which are not subjected to central deletion, are suppressed by dR-hTreg and ultimately undergo peripheral deletion over time.

*Animal Studies.* More than 60 years ago, Main and Prehn used bone marrow infusion following administration of high-dose, lethal total body irradiation (TBI) to achieve skin allograft tolerance in recipient mice. In this experimental setting, semiallogeneic but not isogenic bone marrow infusion consistently permitted donor-specific skin graft acceptance (10). Subsequent studies from Cobbold and colleagues showed that mice treated with T-cell depleting antibodies along with TBI did not reject MHC-mismatched bone marrow grafts and developed donor-specific tolerance (11). These mice exhibited full donor chimerism, i.e. the entire recipient hematopoietic system was replaced by donor cells (donor cells > 98%), so “self” tolerance of donor T cells was achieved. Later studies suggested that incomplete deletional tolerance of these recipient-reactive donor T cells was achieved, reflecting the absence of a self-renewing source of recipient APCs to ensure complete deletion of host-reactive donor T cells in the thymus. Nevertheless, functional tolerance to the recipient was achieved by a combination of mechanisms that involve thymic stromal cells, which are of recipient origin (12, 13) (**Figures 1A, B**).

Several strategies have been studied to reduce the risk of bone marrow engraftment failure and to curtail the impact of myeloablative conditioning regimens that were initially necessary to allow the engraftment of allogeneic bone marrow stem cells. Ildstad et al. reported the engraftment-promoting effects of a cell product termed “facilitating cells” (FC) in mice treated with high TBI doses. Murine FC include a population of CD8α^+^ TCR^-^, but paradoxically CD3^+^, plasmacytoid-precursor dendritic cells and also seem to include populations of B cells, NK cells, granulocytes and monocytes. Murine FCs have been reported to provide survival and homing signals to HSC, induce antigen-specific regulatory T cells (Tregs) and expand IL-10-producing Tr1 cells (14–17). These cells were also reported to be present in human bone marrow (18) and have served as the basis for the proprietary product used in the Northwestern University clinical protocol described below.

*Clinical Protocols.* Investigators from Northwestern University utilized a non-myeloablative conditioning regimen that achieved durable full donor chimerism in humans, attempting to exploit the engraftment-promoting and immunosuppressive effect of FC (19, 20). This regimen builds on the Hopkins protocol that uses post-transplant cyclophosphamide to inhibit GVHD across HLA barriers (21) and includes pre-transplant fludarabine, cyclophosphamide and TBI, which “make space” for HSC engraftment and control anti-donor responses that would otherwise lead to graft rejection (**Table 1**). Kidney transplantation is followed by infusion of a G-CSF +/- plerixafor-mobilized apheresis product treated to retain HSC and FC, as well as a controlled number of donor T cells. While the proprietary method for apheresis product treatment has not been disclosed, the full chimerism achieved in most of these patients, despite non-myeloablative conditioning, suggests a major role for GVH-reactive donor T cells in destroying recipient hematopoietic cells in the bone marrow.

**Table 1:** Tolerance-inducing protocols for kidney transplantation across MHC barriers.

	Northwestern University	Massachusetts General Hospital	Samsung Medical Center
**Type of Chimerism**	Durable Full-Donor	Transient Mixed	Transient Mixed
**Donor Cells**	G-CSF-mobilized HSC (up to ~17×10^6^/Kg) + T cells (~4×10^6^/Kg) + FC (0.5-12×10^6^/Kg). Infused at +1.	Whole BM (2–3×10^8^/Kg)	Whole BM (0.6-2.2×10^8^/Kg, with HSC 0.8-3.2×10^6^/Kg)
**Conditioning (KTx day 0)**	FLU (30mg/m^2^, -5/-4/-3), CYC (50mg/Kg, -3/+3), TBI (200 cGy, -1)	(*NKD03*) CYC (60mg/Kg, -5,-4), siplizumab (0.6mg/Kg, -2/-1/0/+1), TI (700 cGy, -1). (*mNKD03*) NKD03 + ritux (375mg/m2, -7/-2) + pred from 0 to +10 (*ITN036*): NKD03, + ritux (375mg/m2, -7/-2/+5/+12) + pred from 0 to +20 (*TBI-Pilot*): ITN036 + TBI (1.5 Gy, -5/-4) instead of CYC.	(*Protocol-1*) mNKD03 + rATG (1.5 mg/Kg, -1/0/+1) instead of siplizumab + pred up to 3-6 months (*Protocol-2*) Protocol-1 + FLU (15 mg/m2, -6/-5/-4/-3) + rATG (1.5 mg/Kg, +2) (*Protocol-3*) Protocol-1 + FLU (10 mg/m2, -6/-5/-4/-3) + SIR (from month 1) instead of TAC.
**Maintenance IS**	TAC tapered after 1-year protocol biopsy.	CYA (NKD03 and mNKD03) or TAC (ITN036 and TBI-Pilot) tapered after 6-month protocol biopsy.	TAC (or SIR) tapered after protocol biopsy at 1 year. pred tapered and discontinued at 3-6 months.
**Tolerant patients (> 1 year off IS)/transplanted patients**	26/37	7/10	5/8
**Fatal SAEs**	3	0	0
**GVHD cases**	2	0	0
**Graft losses**	2	6 (3 after > 10 years)	2

BM, bone marrow; CYC, cyclophosphamide; FC, facilitating cells; FLU, fludarabine; HSC, hematopoietic stem cells; G-CSF, granulocyte colony stimulating factor; pred: prednisone; rATG, rabbit anti-thymocyte globulins; ritux, rituximab; SIR, sirolimus; TAC, tacrolimus; TBI, total-body irradiation; TI, thymic irradiation.

Out of the 37 patients transplanted, 26 exhibited durable donor chimerism (23 developed full-donor chimerism) and were successfully weaned off immunosuppression after one year from transplant. These subjects showed significantly better kidney function compared to matched controls receiving conventional immunosuppression. Two graft losses due to opportunistic infections were recorded in the first year after transplantation, and one tolerant recipient died due to sepsis. Studies in mice have highlighted that full donor chimeras are somewhat immunoincompetent (22, 23) due to the absence of recipient APCs in the periphery, which are needed to optimally present antigens to T cells that are positively selected by recipient thymic epithelium. Indeed, cytotoxic T cells generated in chimeric mice lacking shared MHC alleles between the donor and recipient are unable to clear virally infected donor cells (24), which thereby serve as a viral reservoir that can result in chronic illness (23). While viral reactivation and other opportunistic infections occurred quite frequently in patients on this study, patients with full donor chimerism nevertheless could be successfully vaccinated after immune cell reconstitution, likely reflecting, at least in part, persistence of immune memory and immunity carried by donor T cells in the hematopoietic cell transplant (25). Additional complications included acute rejection in two patients with transient chimerism that were non-compliant with medications, and one death due to lung cancer. A potentially alarming toxic effect was recorded after a longer observation period: despite the use of post-transplantation cyclophosphamide, two subjects ultimately developed graft-versus-host disease (GVHD). One patient was diagnosed with grade 3 intestinal GVHD and CMV infection that led to a fatal outcome. Although relatively limited in frequency (5% of treated patients), the risk of GVHD in our view outweighs the benefits obtained with approaches based on full donor chimerism for tolerance induction.

### Mixed Chimerism

*Animal Studies.* Mixed chimerism defines a state wherein, unlike full donor chimerism, the host hematopoietic system is not completely destroyed and replaced by the donor’s, and hematopoietic cells of both the recipient and the donor coexist in the bone marrow.

Sharabi and Sachs demonstrated that durable mixed chimerism and tolerance could be induced in mice conditioned with T cell-depleting antibodies, low-dose TBI and thymic irradiation (TI) (26). This method overcomes peripheral and intra-thymic rejection (27) of donor HSCs and facilitates bone marrow engraftment, which in turn provides a durable supply of progenitors that migrate to the thymus, differentiating into lymphocytes and dendritic cells. Tolerance to donor and recipient in these models is achieved *via* intra-thymic negative selection of alloreactive T cell clones, mediated by both donor- and recipient-derived antigen-presenting cells (28, 29) and regulatory mechanisms are notably absent in the long-term tolerance maintenance phase (30). Durable mixed chimerism can also be achieved through co-stimulation blockade combined with bone marrow transplantation, which resulted in anergy and peripheral deletion of donor-reactive clones (31–33). Peripheral tolerance of donor-reactive CD4 and CD8 T cells relied on distinct mechanisms, with a role for NFAT, LAG3, TGFβ, PD1 and recipient CD4 T cells, B cells and MHC class II for the CD8 T-cell anergy followed by deletion (34–38) and a pathway involving CD4 T cell-intrinsic CTLA4 and recipient CD80 and CD86 without regulatory mechanisms, leading to peripheral CD4 cell deletion (32, 39). The caspase 9-dependent intrinsic and cell-extrinsic Fas-FasL apoptosis pathways have both been implicated in clonal deletion in these models (40, 41). Notably, alternative mixed chimerism-based regimens that do not achieve complete deletion of donor-reactive T cells also rely on alloreactive Treg-mediated suppression to induce donor-specific tolerance (42, 43) (**Figure 1C**).

Before human application, non-myeloablative conditioning regimens for the induction of allograft tolerance were tested in non-human primates [extensively reviewed in (44)], a key step to assess the safety and efficacy of these protocols. These experiments underscored that the rate of success and the stability of chimerism induction in primates is considerably lower compared to rodents, partly due to the higher abundance of memory T cells in the former, which are more resistant to conventional T cell-depleting agents (45). The addition of splenectomy (or co-stimulation blockade) and a short course of cyclosporine could partially overcome this barrier in a significant fraction of animals, but mixed chimerism was only transient in all of them (46, 47). Contrary to initial assumptions, tolerance to renal allografts developed in more than 60% of recipients, providing the first proof of principle that durable chimerism is not essential for tolerance induction in primates, thus paving the road to human translation.

*Clinical Protocols.* Mixed chimerism-based approaches to induce tolerance to kidney allografts have been tested at the Massachusetts General Hospital (MGH) in patients with and without hematologic malignancies. Differences between these regimens have been reviewed in detail elsewhere (48), and we will focus our current discussion on patients without malignancy, as these protocols have the highest potential for translation to routine clinical practice in the future.

Initial studies used a non-myeloablative conditioning regimen that included cyclophosphamide, the anti-CD2 T cell-depleting monoclonal antibody siplizumab and thymic irradiation (TI) (**Table 1**) (49, 50). Unprocessed donor bone marrow was infused on the day of kidney transplantation, and subjects also received calcineurin inhibitors and a short course of corticosteroids postoperatively. Pre- and peri-transplant rituximab doses were introduced after evidence of antibody-mediated rejection in one patient and *de-novo* DSA development in 2 additional patients. After this modification, all patients remained immunosuppression-free for the duration of the study. Transient mixed chimerism for up to 3 weeks was induced in all recipients, without evidence of GVHD. Maintenance immunosuppressive drugs were slowly tapered after 6 months in patients with normal protocol biopsy, and the primary endpoint of 24-month immunosuppression-free kidney allograft survival was achieved in 7 of the 10 patients enrolled. Three of these subjects later (at 4 to 7 years post-transplant) experienced chronic rejection or glomerulonephritis recurrence, which led to reintroduction of immunosuppressive drugs and ultimately resulted in graft loss more than 10 years after transplantation. Of note, these patients were successfully retransplanted with conventional immunosuppression, and there were no significant opportunistic infections in any of them.

In parallel with early host T cell recovery, 9 patients unexpectedly developed severe acute kidney injury. Renal histology was consistent with engraftment syndrome, entailing capillary endothelial injury with vascular leak and lympho-monocytic infiltrating cells in peritubular and glomerular capillaries. Renal function normalized in all but 2 recipients, one of whom experienced graft loss due to acute humoral rejection as a consequence of preformed DSA that were undetectable on a pre-transplant ELISA, but were subsequently confirmed by Luminex. In the other patient, acute kidney injury was initially misdiagnosed as rejection and was treated with higher doses of tacrolimus, which triggered thrombotic microangiopathy. Finally, one patient developed severe cellular rejection after a pyelonephritis episode following immunosuppression withdrawal. Protocol biopsies (at 2-8 years) in tolerant subjects showed either completely normal histology or minimal alterations, including focal glomerular basement membrane duplication and mild podocyte foot process effacement (50).

An additional protocol was tested at MGH based on further observations from studies conducted in non-human primates (46, 51). Compared to previous regimens, cyclophosphamide was substituted with TBI to prevent engraftment syndrome. Renal function remained stable in the two patients enrolled, but one did not develop sufficient chimerism to allow immunosuppression weaning. Immunosuppressive drugs were successfully discontinued in the other patient, but were resumed after more than 4 years due to evidence of humoral rejection on a protocol biopsy (52).

Investigators at the Samsung Medical Center initially used a nearly identical protocol to those outlined above, but the anti-CD2 monoclonal antibody siplizumab was substituted with ATG due to local unavailability (52, 53). To curtail the risk of engraftment syndrome, fludarabine and an additional dose of ATG were added in a second protocol iteration, which allowed reduction of the dose of cyclophosphamide. Due to development of BK nephritis, ATG and fludarabine dose was subsequently decreased, and tacrolimus was substituted with sirolimus one month after transplantation. Overall, mixed chimerism was achieved transiently (at least 3 weeks) in all 8 enrolled subjects. Immunosuppression was successfully discontinued for more than one year in 5 patients, even though one of them experienced acute cellular rejection after a respiratory tract infection, which led to reintroduction of tacrolimus.

## Mechanistic Studies in Humans and Methods to Track Tolerance

### Full Donor Chimerism

The mechanism that underlies tolerance to kidney allografts associated with full-donor chimerism hypothetically involves central tolerance of donor T cells to donor antigens, with donor progenitor cells migrating to the recipient thymus, differentiating into antigen-presenting cells and finally mediating negative selection of “self”-reactive donor T cell clones. Bulk functional assays, including mixed-lymphocyte reactions (MLR) and cell-mediated lympholysis (CML), demonstrated donor-specific hyporesponsiveness in tolerant patients. However, the same effect was observed in recipients who exhibited only transient chimerism and developed rejection after immunosuppression withdrawal (54), suggesting that these assays cannot be relied upon to infer a tolerant state. On the other hand, development of full donor chimerism was the single most accurate predictor of tolerance in these patients (54). An intra-graft signature of tolerance was also described for these patients, which was characterized by upregulation of genes involved in B cell regulation and pro-tolerogenic plasmacytoid DC enrichment, as well as the induction of regulatory pathways involved in the control of inflammation and maintenance of tissue homeostasis (55). Overall, however, studies elucidating the mechanism of tolerance in these subjects are currently lacking.

Given the full donor chimerism achieved in these patients, the achievement and mechanism of GVH tolerance is also worthy of investigation. Studies in mouse models discussed above would suggest that *de novo* GVH tolerance might be characterized by a combination of clonal deletion, anergy and regulatory T cell-mediated mechanisms. However, GVH tolerance has not been demonstrated in these patients and the inclusion in the infused product of mature donor T cells that eliminate host hematopoiesis suggests that an ongoing GVH reaction may occur, which has culminated in GVHD in several patients. Whether or not GVH reactions in patients without overt GVHD results in thymic injury and failure to negatively select host-reactive T cells, as reported in murine models (56–59), has not been investigated.

### Mixed Chimerism

The mechanisms of tolerance in protocols based on transient mixed chimerism have been the topic of extensive studies in recent years. Central tolerance is unlikely to be the main mechanism operating in these CKBMT patients, since transient chimerism is likely insufficient to allow long-term thymic repopulation with donor antigen-presenting cells.

Preliminary studies with bulk functional assays were partly inconclusive, since a lack of post-transplant donor-specific responses was observed both in tolerant patients and in the patient who developed acute rejection after immunosuppression withdrawal in the MGH trial (60). Several mechanisms, including T cell anergy and peripheral deletion, could underlie the observed donor-specific hyporesponsiveness, but these assays could not discriminate between them. Nonetheless, these results were extremely informative when compared with those from recipients of bone marrow transplantation conditioned with a similar regimen but without kidney transplantation. In these subjects, donor-specific reactivity reappeared after chimerism was lost, indicating that the kidney allograft is likely to play a pivotal role in tolerance development in CKBMT recipients (61).

The advent of platforms to perform high-throughput sequencing of the TCRβ CDR3 hypervariable region led to the development of novel tools to analyze the T cell alloresponse. We hypothesized that a significant fraction of the donor-reactive repertoire could be identified in a pre-transplant MLR, by sequencing sorted recipient T cells that divided in response to donor stimulation. These sequences were compared with those of sorted unstimulated recipient CD4^+^ and CD8^+^ T cells to define a fingerprint of the anti-donor T cell repertoire. Thresholds for detection were based on a uniform clonal frequency (to normalize for sample size variability over time) and on a minimal fold-expansion (to avoid capturing highly abundant but not specifically donor-reactive clones), while computational methods were used to account for sorting errors (62). This fingerprint was then longitudinally compared with samples obtained at different post-transplant time points to track circulating donor-reactive clones over time. Both tolerant and non-tolerant patients, as well as kidney transplant recipients under conventional immunosuppression, had considerable repertoire turnover, reflecting the use of T cell depleting agents in the conditioning regimens. However, all tolerant patients analyzed displayed a progressive and specific reduction in both donor-reactive CD4^+^ and CD8^+^ T cell clones, whereas no significant change was identified in the non-tolerant patient (60), and conventional kidney transplant recipients showed expansion of CD4^+^ T clones. These results suggest that clonal deletion is involved in the development of tolerance and may serve as a marker to identify patients amenable to safe immunosuppression weaning. Conversely, T cells in the non-tolerant patient were probably anergic, but were re-activated after immunosuppression withdrawal by the infective episode, thus precipitating acute rejection.

The existence of a suppressive mechanism in these patients was initially suggested by re-emergence of anti-donor responses in bulk functional assays performed with Treg-depleted samples from the first post-transplant year. However, samples obtained at later time points failed to show a similar response, suggesting that suppression could be relevant only as an early mechanism (63). Consistent with this hypothesis, limiting dilution assays conducted after the first post-transplant year failed to show an increase in response at higher dilution, which usually indicates the presence of suppressive cells at a lower frequency than responder cells (60).

Phenotypic analysis of circulating mononuclear cells in tolerant patients identified an early expansion of Tregs (80% of CD4^+^ T cells during the first week) with evidence of peripheral proliferation, possibly recent thymic emigration and, in one patient, conversion from conventional T cells (64). Expression of CD45RA declined after two weeks from transplant (64), suggesting that previously resting Tregs acquired an activated phenotype (65). The presence of a highly demethylated FoxP3 Treg specific region, an epigenetic hallmark of stable Tregs, confirmed the results from phenotypic data.

Subsequent studies demonstrated that the anti-CD2 monoclonal antibody siplizumab could induce costimulation blockade and T cell depletion, but selectively spared Tregs and promoted the expansion of alloreactive Tregs *in vitro* (66). *In vivo*, this process may be further amplified by the lymphopenia-driven expansion state that follows global T cell depletion. Interestingly, siplizumab predominantly reduced the frequency of effector memory T cells, which express the highest CD2 levels among T cell subsets (66, 67). This additional effect may be relevant for tolerance induction, since cross-reactive memory T cells are abundant in humans, and constitute a barrier to the establishment of chimerism and tolerance. Indeed, these cells are more resistant to depletion with ATG, depend less on costimulatory signals and are less susceptible to Treg-mediated suppression (68).

By using the same sequencing approach detailed above, we interrogated donor-reactive sequences that mapped to the unstimulated sorted Treg pool, but these sequences were detected at a very low frequency due to the low numbers of Tregs in the circulation. The method was therefore optimized by expanding the donor-reactive Treg pool with activated donor B cells instead of performing a conventional MLR. Expansion of donor-specific Tregs with activated donor B cells greatly increased the number of unique donor-specific Treg sequences identified and the specificity and potency of these cells in suppressing anti-donor responses was markedly increased, demonstrating that truly donor-specific Tregs were enriched in this repertoire. Using this method of pre-transplant donor-specific Treg repertoire identification, tolerant patients were found to display significant expansion of donor-specific Tregs at 6 months from transplantation, while the single non-tolerant subject did not (69). This study also showed that the majority of expanded Tregs in tolerant subjects mapped to the pre-transplant unstimulated Treg pool rather than conventional T cells, suggesting that expansion of pre-existing Tregs rather than induction of donor-specific Tregs was the major mechanism for increased donor-specific Tregs in these patients.

Overall, these data indicate a central role for early Treg-mediated suppression in the development of tolerance in combined kidney bone marrow transplantation. It could be speculated that prolonged stimulation of donor-reactive T cell clones by graft antigens under constant restraint by Tregs might mediate anergy and subsequent peripheral deletion of these cells. This suppressive effect loses potency over time as gradual clonal deletion of donor-reactive T cells eliminates the alloresponse needed to maintain expanded donor-specific Treg populations (**Figure 1D**).

## Future Perspectives

Even though tolerance induction has been achieved in humans through chimerism development, these regimens still need to be refined before they can be translated to routine clinical practice. We believe that the ultimate aim will be to develop a protocol capable of reproducibly inducing tolerance through durable mixed chimerism.

Albeit progressively refined over the course of the last decades, conditioning regimens still bear potentially significant systemic toxicity, which results in both short- and long-term clinically relevant complications. The development of costimulation blockers and other novel drugs targeting specific cell populations and molecular moieties could help to refine conditioning regimens further, thus limiting side effects. Avoidance of engraftment syndrome observed in current regimens represents a realistic short-term goal, which may be achieved with revised protocols in the near future. Studies in animal models and humans have outlined that several mechanisms for tolerance coexist, and future strategies may exploit this knowledge to induce a more robust tolerant state. A future, intriguing possibility to promote durable chimerism without increasing the risk of GVHD is represented by peri-transplant infusion of *ex-vivo* expanded recipient Tregs. Administration of polyclonal Tregs was able to induce mixed chimerism in mice in the absence of cytoreductive therapy (70), and promoted more durable mixed chimerism and tolerance, that permitted delayed kidney transplantation without immunosuppression, in primates treated with non-myeloablative conditioning (71).

Reproducibility in humans remains a key issue of translational research in transplantation, especially in the context of tolerance trials, where a universally accepted and validated biomarker of the tolerant state has been lacking so far. The newly developed methods based on TCR sequencing to track donor-reactive T cell/Treg clones deserve further exploration as a tool that may be useful for the identification of patients amenable to safe immunosuppression withdrawal in a personalized manner. Furthermore, this approach has considerable potential to further identify the role of and elucidate mechanisms of host-vs-graft and graft-vs-host reactivity and tolerance, respectively, in recipients of hematopoietic cell transplantation for the purpose of allograft tolerance induction.

Tolerance studies will be also pivotal to pave the way to clinical xenotransplantation, considered to be the next frontier in solid organ transplantation due to its potential to overcome the severe shortage of human organs. Murine models have shown that mixed chimerism induction can promote tolerance to xenografts through several concomitant mechanisms, including deletion of xenoreactive B cells (72–75) with disappearance of natural antibodies to xenoantigens, as well as tolerization of xenoreactive T (76) and NK cells (77). These results have been replicated by induction of porcine mixed chimerism in immunodeficient mice with human immune systems (78–81). However, immune barriers to xenogeneic mixed chimerism induction are considerably greater than those to allogeneic chimerism, particularly due to the rapid destruction of porcine cells by human macrophages (82, 83), which can be at least partially overcome by the introduction of a human CD47 transgene into the pig (84–86). Current protocols will need to be optimized before clinical translation can be safely attempted.

## Author Contributions

MP and MS jointly wrote the first draft of the manuscript and revised its content critically. All authors contributed to the article and approved the submitted version.

## Funding

This work was supported by a grant from the American Society of Transplantation Research Network. The work was also supported in part by NIH grants (ROI #AI084074), Immune Tolerance Network contract (N01 AI15416) and Immune Tolerance Network Award (UM1 AI109565).

## Conflict of Interest

The authors declare that the research was conducted in the absence of any commercial or financial relationships that could be construed as a potential conflict of interest.

## Publisher’s Note

All claims expressed in this article are solely those of the authors and do not necessarily represent those of their affiliated organizations, or those of the publisher, the editors and the reviewers. Any product that may be evaluated in this article, or claim that may be made by its manufacturer, is not guaranteed or endorsed by the publisher.
